# Update on Safety and Efficacy of HPV Vaccines: Focus on Gardasil

**DOI:** 10.22088/IJMCM.BUMS.10.2.101

**Published:** 2021-09-01

**Authors:** Monica Soliman, Ololade Oredein, Crispin R. Dass

**Affiliations:** 1 *Curtin Medical School, Curtin University, Bentley, WA 6102, Australia.*; 2 *Curtin Health Innovation Research Institute, Curtin University, Bentley, WA 6102, Australia.*

**Keywords:** HPV, Gardasil, vaccine, safety, Cevarix

## Abstract

The human papillomavirus (HPV) is a highly contagious and prevalent virus that is primarily sexually transmitted. The Gardasil® quadrivalent vaccine, the Cevarix® bivalent vaccine and the Gardasil® 9 nonavalent vaccine were developed to prevent the spread of HPV as well as the incidence of its associated diseases. The aim of this mini-review is to critically analyze the safety and efficacy of both the Gardasil vaccines. A literature search was conducted on ProQuest, MedLine, Science Direct and Scopus databases. More than hundred articles were scanned, and from this, 38 most relevant papers involving human studies across several countries were closely reviewed. The literature deems the Gardasil® HPV vaccines to be safe and efficacious. Due to the novel nature of these vaccines, long-term efficacies, as well as their associated long-term adverse effects, are yet to be confirmed. Of some concern was the finding that a majority of these studies disclosed minor to major involvement with the vaccine manufacturers, and the inhibitory cost of use in developing nations. Gardasil is largely considered safe to use. However, considering that these vaccines are predominantly indicated for children, further comprehensive, impartial, and long-term studies are needed to critically assess safety and efficacy.

## Introduction

The human papillomavirus (HPV) is a highly contagious and prevalent virus that is primarily sexually transmitted (1-3). It is composed of double stranded DNA, containing two subunits: L1 and L2, the former of which is the sole target of modern HPV vaccines (4). HPV affects the mucosa and skin of aerodigestive and anogenital tracts in both men and women, causing various cancers and neoplastic lesions of varying severity (1). HPV is the cause of 5% of cancers worldwide (4), including cervical, anal, vulvar, penile, oropharyngeal, and vaginal (5, 6). Cervical cancer has resulted in at least 250,000 deaths per year and is rising, especially in developing countries, where nearly 80% of cervical cancer-related deaths have been recorded (1). Yet, the cost of the vaccines in these countries has surpassed manufacturing costs by even 10-fold in those same countries (6).

**Fig 1.**
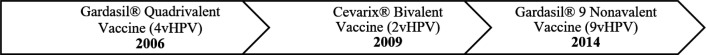
Timeline of the United States Food and Drug Administration (FDA) approval of HPV vaccines currently available on the market

These vaccines, available in the market since 2006 (Figure 1), are composed of recombinant HPV proteins that form virus-like particles (VLPs) which act as neutralising antibodies to eliminate the viral effect of the HPV genotypes found in the vaccine. This allows for the body’s development of endogenous antibodies against the genotypes (Table 1), providing a mechanism to develop long-term immunity against the virus. However, these vaccines do not have the capability to treat any pre-existing HPV infections or related conditions. Vaccine efficacy is difficult to determine due to the lengthy bout of time from viral exposure to disease onset, thus eluding to the possibility of undetectable flaws in the effectiveness and safety of these vaccines. 

The development of these vaccines (Figure 1) allowed for a multi-faceted approach to their mechanism of immunity. Foremost, as previously mentioned, the neutralising antibodies play the focal role in defence against the various virus genotypes (Table 1), rather than cell-mediated immunity. The vaccines readily generate these neutralising antibodies, which continue to deactivate the virus in elevated and enduring titres. This phenomenon is typically limited to genotypes found in the vaccine and is minimally observed in cross-type protection of other genotypes, although studies vary in degree and duration of cross-protection, as will be discussed later. Moreover, these vaccines allow for negligible risk of viral transmission and produce significant antibody-mediated suppression due to the virus’ innate vulnerability. 

The Gardasil® quadrivalent vaccine was developed by Merck and Co., Inc. and protects against HPV6 and HPV11, responsible for genital warts (3,5) and recurrent respiratory papillomatosis (RRP) (3), as well as HPV16/18 (5). The Cevarix® bivalent vaccine, manufactured by GlaxoS-mithKline Biologicals, was developed to target the two most problematic strains (HPV16/18) (7). The Gardasil nonavalent vaccine, the most comprehensive and latest to be available in the market, is active against HPV 6/11/16/18/ 31/33/ 45/52/ 58, which are responsible for 90% of cervical cancer cases worldwide (3,7). 

The aim of this review is to critically analyze the safety and efficacy of the Gardasil® vaccine, both the quadrivalent and nonavalent varieties. Safety would be characterized as the adverse effects associated with the vaccinations whereas efficacy would be illustrated via various defined end-points. We performed a multi-study review of current literature on both variations of the Gardasil vaccine.

**Table 1:** Licensed HPV vaccines

	**Gardasil® ** **Quadrivalent**	**Cevarix® ** **Bivalent**	**Gardasil® 9** **Nonavalent**
**Manufacturer**	Merck & Co.	GlaxoSmithKline	Merck & Co.
**Targeted ** **HPV genotypes**	6, 11, 16, 18	16, 18	6, 11, 16, 18, 31, 33, 45, 52, 58
**Adjuvant**	Amorphous aluminium hydroxyphosphatesulphate	3-O-desacyl-4’-monophosphoryl 300 lipid A and aluminium hydroxide	Amorphous aluminium hydroxyphosphate Sulphate
**Year Marketed Internationally**	2007	2011	2015

**Search strategy**

A literature search was conducted on ProQuest database for search terms *Gardasil *and *safe *or *efficacy of Gardasil*, with the following search limitations: *peer reviewed, full text, scholarly journals (not commentary, review, literature review, editorial, correspondence), publication date: after 2014, English* and this yielded 646 results. Another search was conducted on MedLine for *Gardasil*, while limiting to *humans, English language, full text* and found 56 research articles. Science Direct was searched using *Gardasil*, with limiters: *year(s): 2014-2019, no review articles*, and obtained 493 results. Scopus was also employed to search *Gardasil *and *vaccine *and *safe *or *effect *or *immune *or *outcome *or *adverse, *which highlighted 214 scholarly journal articles. Among those found, we selected literature most relevant to our aim, particularly original studies from the previous five years. 

We reviewed 30 papers including two involving animal studies and 28 involving human studies. Among the human studies, participants were selected from several countries including Canada, Australia, USA, Mexico, Mongolia, Italy, Denmark, Sub-Saharan Africa, UK, India, Finland, France, Hong Kong, Singapore, among others. Included in the human studies assessed were those focused on immune-compromised and pregnant women. The participants in the various studies included men, women, and children, from 9 years of age until adulthood. 

**Safety**

Safety, as defined in this review, is characterised by presence of adverse effects following immunisation (AEFI) in participants. A serious adverse effect is defined as any event that results in death, life-threatening experience, hospitalisation or prolonged hospitalisation, significant disability and/or congenital abnormalities. These include, but are not limited to, data compiled from non-manufacturer reports, Vaccine Adverse Event Reporting System (VAERS), hospital medical records, gynaecological departments, and vaccination centres. 

**Safety in animals**

A study was performed on female mice to determine the safety profile of the Gardasil® quadrivalent vaccine when compared to its aluminium adjuvant. Safety was determined via behavioural tests including a forced swimming test (FST) conducted three months post-administration of the vaccine. Inbar *et al.* found statistically significant differences in the performance of the aforementioned test between the group that received aluminium adjuvant and those that received the quadrivalent vaccine (8). They also measured serum antibody levels in the mice, one month after their administration with the quadrivalent vaccine or the aluminium adjuvant. They found elevated levels of antibodies targeting mouse brain phospholipids and mouse brain protein extracts (8). This may be explained by an amino acid sequence similarity between the antigen present in the vaccine and those in body cells, such as proteins. The structural similarity may be problematic and result in unintentional antagonism of these endogenous cells, leading to health crises. This may explain how the majority of adverse effects reported post immunisation seem to be of a neurological nature. This reinforces the need for further caution in regards to mass immunisation due to the sensitive nature of potential cross-species reactivity to these vaccines. 

Research from Wise *et al.* was conducted to examine the exposure of the nonavalent vaccine in a population of Sprague-Dawley rats (9). The study aimed to evaluate the toxicity in a sample of 200 female and male rats, half of which received the Gardasil® 9 vaccine while the other half, or control group, received a phosphate buffered saline (PBS) solution. They observed injection associated muscle fibre degeneration, described as swelling of myofiber tissue, sarcoplasm fragmentation resulting in inflammation, and consequently, the presence of inflammatory mediators. Admittedly, caution must be taken when extrapolating animal data to humans, and further testing is required. 

**Safety in females**

Table 2 is a summary of studies, mainly clinical, testing HPV vaccines. A study by Garland *et al.* found that participants who received the 9vHPV vaccine reported one or more adverse effects more often than those in the placebo group who received a saline injection, with 95.9% and 75.1% participant reports, respectively. Moreover, the number of participants in the vaccine group who recorded a body temperature above 37.7^o^C was more than double of that in the placebo group. Furthermore, multiple studies concur that participants receiving the nonavalent vaccine reported more injection site reactions than its quadrivalent counterpart (10,19, 32). They concluded, however, that the nonavalent vaccine was well tolerated. 

A randomised, double-blind study of 9-15-year-old girls was performed to investigate the safety profile of the quadrivalent and nonavalent Gardasil® vaccines (32). The participants were allocated to two groups, each receiving a three-dose regimen of either vaccine. The vast majority of participants reported at least one adverse effect in the 9vHPV group and the 4vHPV group (93.3% and 90.3%, respectively). The most common systemic adverse effects in the 9vHPV group were nausea (3%), fever (5%), upper abdominal pain (1.7%), oropharyngeal pain (2.7%), and headache (11.4%). Whereas in the 4vHPV group, nausea (3.7%), fever (2.7%), headache (11.3%), upper abdominal pain (1.3%), and fatigue (2.7%) were the most frequent. 

An Italian case-based study examined post-HPV immunisation reports by employing a systematic approach to a causality assessment algorithm (30). They found 19 cases of interest from 2008 to 2016 that described serious systemic adverse effects following the administration of the bivalent or quadrivalent vaccine. They concluded that only half of these cases were related to the vaccine itself, and that further research is imperative to design a better system of reporting and determining the causes of such serious reactions. A United States Vaccine Adverse Event Reporting Systems (VAERS) recorded 55,356 individual case safety reports (ICSRs) regarding HPV vaccines from 2007 to 2017 (2). It reported more than 6,640 serious AEFI (12%) and less than 1% fatalities, up to 120 days following the vaccine administration (2).

Lui *et al.* performed a widespread meta-analysis of all adverse effects reported following HPV immunisation in Alberta, Canada from 2006 to 2014 (23). They observed 37.4 AEFI reports for every 100, 00 doses of vaccine administered, none of which resulted in fatalities. Another Canadian study collected the AEFI reports in Ontario from September 2007 to December 2011, the first four years following the implementation of a school-based HPV program (17). Among the 152 reported adverse events, the majority were local injection site reactions (20%), rash (22%), non-anaphylactic allergic reactions (25%), and other severe or unusual events (26%) (15). They concluded that the incidence of AEFI were quite low and concurrent with those reported elsewhere (17, 23).

An observer-blinded study was conducted to determine the safety profile of a two or three dose schedule of the quadrivalent vaccine compared to two doses of the bivalent vaccine (2). 4% of the participants reported at least one serious adverse effect, half of which were from the group receiving two doses of the bivalent vaccine while the remainder were from the groups receiving two or three doses of the quadrivalent vaccine. However, the investigators of this study determined that none of these serious adverse effects were vaccine related. 

**Table 2:** Summary of selected literature, including vaccine manufacturer(s) involvement

**Study ** **reference**	**Number of ** **participants**	**Vaccine(s)**	**Involvement of vaccine manufacturer(s)**	**Summary of finding(s)**
(1)	9,111	4vHPV	One author received grants and was on the advisory board at MC	*
(2)	55,356	2vHPV 4vHPV 9vHPV	---	*
(4)	2,520	9vHPV	Funded by MC and grants from GSK and MC	9vHPV is highly immunogenic and should be administered to both men and women (*).
(5)	111,804	4vHPV	---	HPV associated cervical neoplasia risk was lower in women vaccinated under the age of 18.
(7)	34	4vHPV	Research grant from MC	4vHPV was highly immunogenic and well tolerated (*).
(8)	76 (mice)	4vHPV	---	HPV antigens found in the vaccines may inadvertently also target brain antigens
(9)	300 (rats)	9vHPV	All authors are current/ former employees of MC; laboratory methods conducted at MC	*
(10)	198	2vHPV 4vHPV	SP (partnered with MC) provided vaccine	Cross-neutralising antibodies may play a role in cross-protection and immunogenicity against HPV.
(11)	91		Previous employment of authors by MC	4vHPV was immunogenic in the HIV-infected population.
(12)	935	4vHPV 9vHPV	Funding, support, contracted authors and executed by MC and GSK	9vHPV was generally well tolerated; serious vaccine adverse effects were rare.
(13**)**	92	4vHPV	---	4vHPV had similar immunogenic effects in HIV infected population compared to non-infected (*).
(14)	371	2vHPV 9vHPV	---	Mixed schedule dosing (2vHPV then 9vHPV) offers similar protection compared to two doses of the 9vHPV.
(15)	150	4vHPV	Funding and research grants from MC	4vHPV is highly immunogenic and safe in mid-adult aged men
(16)	198	2vHPV 4vHPV	---	Neutralising antibodies were detected more than 7 years after the initial vaccine administration; 2vHPV serum titre values were higher than those of 4vHPV.
(17)	>21,000	4vHPV	---	*
(18)	13,306	2vHPv 4vHPV	Authors were previously employed/contracted and members of advisory boards at GSK and SP	HPV vaccination is expected to reduce the incidence of high-grade cervical diseases in the UK.
(19)	14,215	9vHPV	Funded by MC; previous/ current employment, grants, consultancies and other associations with MC, GSK, SP	*
(20)	534,580	2vHPV 4vHPV	---	Adverse effects reported may have been pre-existing prior to vaccine administration.
(21)	98,561	2vHPV 4vHPV	Funding by GSK; grants and employment by MC and GSK	*
(22)	1,075	2vHPV 4vHPV	Funding, design, development, employment of authors and publication by GSK	*
(23)	195,270	2vHPV 4vHPV	---	Post-immunisation adverse effects were very low (*).
(24)	59	4vHPV	Laboratory testing and support by MC; research funding and advisors for MC and GSK	Immunocompromised children had an immune response to vaccination regardless of age or cause of immunosuppression.
(25)	59	4vHPV	Research funding and advisors for MC and GSK; serologic analysis conducted by MC	Three dose schedules were shown to be immunogenic 5 years post immunisation in immunocompromised children.
(26)	147	4vHPV	---	No safety concern among pregnant women receiving the vaccine.
(27)	250	4vHPV	Funding, stock holders, current/former employment of authors by MC	4vHPV was well tolerated and highly immunogenic.
(28)	17,729	4vHPV	Funding by MC and GSK	A single 4vHPV dose is immunogenically equivalent to two or three doses (*).
(29)	1,051	4vHPV	Current/former employment of authors and grant(s) by MC and GSK	Unvaccinated pregnant women had a higher incidence of HPV16/18 compared to vaccinated pregnant women.
(30)	19	2vHPV 4vHPV	---	Reporting systems require a more scrupulous focus on causality'
(31)	500	4vHPV 9vHPV	Current/former employment of authors by MC, SP and GSK; funding, study design, collection analysis and interpretation of data by SP	The efficacy and safety profiles of both vaccines were comparable (*).
(32)	600	4vHPV 9vHPV	---	The efficacy and safety profiles of both vaccines were comparable (*).

--- indicates no involvement or contributions by vaccine manufacturer(s). MC: Merck & Co; GSK: GlaxoSmithKline; SP: Sanofi Pasteur MSD; 2vHPV: bivalent vaccine; 4vHPV: quadrivalent vaccine; 9vHPV: nonavalent vaccine. * Results concurrent with previously established safety and/or efficacy profile.

Furthermore, a meta-analysis was performed on data obtained from the Danish National Health Insurance Service Register in regards to HPV vaccinated women born in 1974 to 2003 (20). They focused their study on the frequency of hospital contacts up to 5 years prior to vaccine administration, and determined that it is often not possible to determine whether an adverse experience was caused by the vaccine itself or due to a prior medical issue. Mugo *et al.* performed a double blind study on female adolescents and adults from Ghana, Kenya and Senegal to assess the safety of a three dose regiment of quadrivalent vaccine (27). They determined the most common adverse effects to be local injection site reactions, 71.6% on average among all three vaccination groups, when compared to an incidence of 47.4% in a control placebo group. They concluded that the quadrivalent vaccination was generally well tolerated in this population. 

Pregnant women are a subset of the human population that has often been neglected when considering the use of vaccines. The quadrivalent vaccine has a pregnancy category B, indicating that limited safety data is available (26). Another study performed a search of US VAERS from June 2006 to December 2013 to find reports concerning pregnant women using the automated system. Among 147 reports found, approximately 18% involved pregnancy specific adverse effects including 15 cases of spontaneous abortion (10.2%). Their review of these reports concluded that there were no safety concerns regarding pregnant women or their infants after receiving the vaccination. Thus, additional research is essential prior to implementing a widespread vaccination program.

**Safety in males**

A study was conducted on heterosexual and homosexual men to ascertain the safety of the quadrivalent vaccine compared to women (15). Males in this mixed gender study reported fewer adverse effects than women in the same study. This may be due to the reluctance of men to report such effects as a result of societal masculine pressures or norms. The reported AEFI were mostly injection site reactions of mild to moderate pain intensity. Likewise, research was conducted to evaluate the safety of the nonavalent vaccine in men. Approximately 76% of male participants reported one or more adverse effects compared to 89% of females in the same study. The most commonly reported AEFI for males were pyrexia (2.4%), headache (7.3%), and injection site reactions (67.6%) such as erythema, swelling, pain and pruritis. They concluded that the vaccine was generally well tolerated and no vaccination induced serious adverse effects were observed (4, 15). 

A phase II clinical study was conducted to compare the safety of the quadrivalent and nonavalent HPV vaccines in men from Germany, the Netherlands and Belgium (31). All participants received three doses of the quadrivalent or nonavalent vaccines on day 1, month 2, and month 6. The adverse effects were comparable in both groups, with 81.5% and 79% in the nonavalent and quadrivalent groups, respectively. However, there were more local injection site reactions in the group receiving the nonavalent vaccine, but this may be due to the higher dose of virus like protein (VLPs) and adjuvants in the 9vHPV compared to the 4vHPV. Correspondingly, the safety and tolerability profiles of both vaccines were analogous. 

**Safety in immunocompromised persons**

Immunocompromised persons are especially prone to infections that are viral or bacterial in nature. Diseases such as HPV are much more prevalent in immunocompromised populations due to their weakened or impaired immune systems (7, 11, 13, 24, 25). 

The use of vaccines to prevent HPV related diseases in this population has been disputed and research has been conducted to perform risk-benefit analyzes. In a study composed of 34 female participants with systemic lupus erythematosus (SLE), the reported adverse effects were comparable to healthy women receiving the vaccine. There was no associated increase in hospitalisations or emergency situations post-immunisation, and vaccination was considered as generally safe (7). 

Unlike with influenza vaccines, the administration of the 4vHPV has not caused any transient demyelinating disorder. Thus, the vaccine was well tolerated and they did not encounter SLE symptomatic flare ups or production of autoantibodies.

In a study conducted on HIV infected persons, the effect of the quadrivalent vaccine was observed and compared to non-HIV infected persons (13). The quadrivalent vaccine was generally well tolerated, many participants reported local pain at injection site: 18.8% of HIV negative and 32.6% of HIV positive participants (11, 13). 

These results were concurrent with previous data, specifically similar for HIV positive participants. Nonetheless, results in this study were lower in HIV negative participants than in other studies (13). This indicates inconsistencies, and suggests that further research is required. 

**Efficacy**

Research on the efficacy of the Gardasil® vaccine has been widespread as shown in Table 3. To determine efficacy, the use of cancer diagnoses as endpoints is unethical and unachievable due to the long lag time from infection to cancer, which may take 7-10 years or more. Thus, each study determined their own endpoint to evaluate efficacy. For instance, some studies applied an incidence-based approach. Other studies also assessed the efficacy of these vaccines using novel methods, such as the presence of anti-HPV antibody titres and vaccine effectiveness (VE). Notably, the nonavalent and quadrivalent Gardasil® vaccines were deemed immunogenically similar in their action against HPV6/11/16/18 in both males and females, according to the research we assessed. Table 3 illustrates the individualised endpoints and findings of these studies. 

**Table 3:** Various efficacy endpoints and summaries of findings determined by the selected literature

**Study ** **reference**	**Vaccine(s)**	**Efficacy endpoint(s)**	**Summary of finding(s)**
(1)	4vHPV	HRHPV detection rates and two-sided p-values were adopted to compare HPV prevalence in the vaccinated and unvaccinated groups	Vaccine targeted HPV detection rates have significantly decreased among Mongolian women who received the 4vHPV vaccine. Overall, there was no significant difference in prevalence of HRHPV for the vaccinated (37.2%) and unvaccinated (41.8%) groups. Yet, among the most carcinogenic strains, HPV16/18/45, they found the prevalence of HPV in the vaccinated group (4.8%) was much less than the unvaccinated group (17.2%).
(4)	9vHPV	Serum samples were tested for the presence of anti-HPV6/11/16/18/31//33/45/52/58 and measured using cLIA; seropositivity was determined if anti-HPV serum levels were ≥ 30, ≥ 16, ≥ 20, ≥ 24, ≥ 10, ≥ 8, ≥ 8, ≥ 8, or ≥ 8 mMU/mL, respectively	Another study involving male participants found that the GMTs for heterosexual men (HM) were non-inferior to their female counterparts.9vHPV vaccination seems less efficacious (weaker antibody response) in men who have sex with men (MSM) than HM. This was illustrated in a higher GMTs among all nine HPV types in HM than MSM.
(5)	4vHPV	Cytology results were obtained at cervical screening and three year absolute and RR with 95% of ≥ CIN2; results were compared in those who had been vaccinated and unvaccinated (reference group)	Correspondingly, the entire vaccination process has changed the clinical interpretation of cervical screening results. Three-year risk analyzes were performed and determined to be 5.26% and 0.99% for ≥ CIN2 and ≥ CIN3, respectively, in women that were vaccinated before 18 years of age (95% CI). Conversely, unvaccinated women were found to have a risk of 10.89% and 3.7% for ≥ CIN2 and ≥ CIN3, respectively. The authors of this study, consequently, recommend cervical examinations to be performed at a younger age.
(9)	4vHPV	VE was based on the prevalence of HPV in the vaccinated and unvaccinated pregnant women; it was calculated as VE = 1 - OR	An exploratory study into a cohort of pregnant women was performed in Montreal from 2010-2016 to determine the effectiveness of the quadrivalent vaccine. They concluded that the incidence of HPV16/18 was significantly higher among unvaccinated pregnant women (7.2%) compared to vaccinated pregnant women (1.3%). They also reported a statistically significant vaccine effectiveness score (86.1%), adjusted for age and number of sexual partners in the past year, in those that were vaccinated.
(10)	2vHPV 4vHPV	Genital swabs and serum were tested for the presence of anti-HPV16/18/31/45 antibodies and analyzed by VLP ELISA to obtain GMT which was normalised to the total IgG present in the sample; titres were deemed neutralising at ≥ 20	Draper *et al.* concluded that among the GMT anti-HPV16 (146,979 and 45,220; P<0.001) and 18 (81,434 and 17,907; P<0.001) antibody titres detected, Cevarix® was more efficacious than Gardasil®, respectively. Similarly, the levels for Cevarix® seemed to be higher than that of Gardasil® in regards to the non-vaccine types HPV31/45 as well, with 356 and 124 (P<0.001) and 35 and 13 (P<0.001), respectively.
(11)	2vHPV 4vHPV	Anal and cervical swabs were obtained and anti-HPV antibodies were measured using a multiplex pseudovirion binding assay to determine HPV type-specific IgG antibodies	In one study, the participants were divided into two groups, each being administered with the bivalent or quadrivalent vaccines. Among the male and female HIV positive participants, the bivalent appeared to cause 100% seroconversion for both HPV16/18. Whereas, the quadrivalent showed seroconversion for HPV16 in men (100%) and women (90%) and HPV18 in men (63%) and women (91%) at much lower rates.
(14)	2vHPV 9vHPV	Serum samples were tested for the presence of anti-HPV6/11/16/18/31//33/45/52/58 and measured using multiplex direct IgG ELISA; seropositivity was determined if anti-HPV serum levels were 0.1 AU/mL, 0.1 AU/mL, 0.5 IU/mL, 0.4 IU/mL, 1.3 AU/mL, 2.5 AU/mL, 0.7 AU/mL and 1.2 AU/mL, respectively	In a mixed-gender mixed-vaccine scheduled study, girls and boys were subjected to either two doses of 9vHPV vaccine or a mixed dose of 9vHPV and 2vHPv. They found that anti-HPV16/18 GMTs were higher in those that received the 2vHPV vaccine than those who received two doses of the 9vHPV vaccine; though, the remaining HPV subtype GMTs (6/11/31/45/5/58) were all higher among those who received two doses of the 9vHPV vaccine.
(15)	4vHPV	Anti-HPV antibodies were measured using cLIA; antibody levels were reported as mMU/mL	In a study conducted on men, participants from Mexico exhibited lower anti-HPV18 antibody responses than those residing in the USA, with 286.5 and 314.8 respectively. However, the anti-HPV16 antibody titres were the same, 45 times higher at 7 months post immunisation than on day 1. They also found that vaccine efficacy was equivalent among mid-aged men (27-45 years) and younger men, for whom the vaccine is clinically indicated.
(16)	2vHPV 4vHPV	Serum was tested for the presence of anti-HPV16/18/31/45 antibodies and analyzed by VLP ELISA to obtain GMT	The authors continued their research and published corroborated results six years later (8). Neutralising antibodies were detected more than 7 years after the initial vaccine administration. They found that the Cevarix® titres continued to be higher than its counterpart, Gardasil®. This may suggest that the bivalent vaccine may be more effective than the quadrivalent vaccine yet, more research is required.
(18)	2vHPV 4vHPV	Cervical sample obtained and was analyzed via an HPV PCR-ELISA method using HRHPV and LRHPV probes; results were then genotyped to determine prevalence and compared to HPV vaccine clinical trial data	A UK based study inferred that the Gardasil® vaccine may have prevented up to 33.2% of their population’s cases of HPV16/18 unrelated cervical intraepithelial neoplasia grade 2 or more severe diagnoses compared to Cevarix® (47.1%). Nonetheless, they deemed these results not statistically significant.
(19)	9vHPV	Serum and cervical samples were analyzed using the Bethesda System-2001 to primary endpoints: incidence of high-grade cervical disease*, vulvar diseaseº and vaginal disease• associated with HPV31/33/45/52/58 & non-inferiority of anti-HPV 6/11/16/18 GMT; VE = 100 x (1 – 9vHPV / 4vHPV incidence rate)	In a large scale, randomised, double-blind trial, the incidence of high grade cervical, vulvar and vaginal disease associated with HPV31/33/45/52/58 was found to be 0.5 cases per 10,000 persons among those that received the 9vHPV vaccine compared to 19 cases per 10,000 persons in those that received the 4vHPV vaccine. According to these findings, the researchers calculated a 97.4% VE rating with a 95% CI. Furthermore, they found non-inferior HPV6/11/16/18 GMTs in the 4vHPV versus 9vHPV up to 3 years post-vaccination. They concluded that the 9vHPV could provide more comprehensive coverage and should be implemented worldwide.
(21)	4vHPV	Cervical samples were obtained and analyzed for incidence of ICC and CIN3+; VE = 1 – incidence rate among vaccinated / incidence rate among unvaccinated participants	A study conducted discovered 75 CIN3 and 4 ICC cases among unvaccinated women while only 4 CIN3 cases among the vaccinated cohort, up to 10 years after they received the vaccine. VE for HPV16/18 was calculated to be 27%. However, their results were 22% and 100% for HPV16 and HPV18, respectively.
(24)	4vHPV	Serum samples were analyzed using cLIA; seropositivity was determined at GMT of HPV6/11/16/18 as 20 mMU/mL, 16 mMU/mL, 20 mMU/mL and 24 mMU/mL, respectively	A two-part series studied the effect of the 4vHPV vaccine on immunocompromised persons. Seven months after the first dose, participant seroconversion rates were 93.3%, 100%, 100% and 88.9% for HPV6/11/16/18, respectively.
(25)	4vHPV	Serum samples were analyzed using cLIA and total IgG assays; seropositivity was determined at GMT of HPV6/11/16/18 as 20 mMU/mL, 16 mMU/mL, 20 mMU/mL and 24 mMU/mL, respectively	The follow up study tested the participants’ serum levels 60 months post-immunisation and found a seroconversion rate of 86.5%, 89.2%, 89.2% and 91.9% for HPV6/11/16/18, respectively. These studies propose that immunocompromised persons should undergo immunisations, such as HPV vaccines, in order to develop immunity and prevent the onset of associated diseases.
(27)	4vHPV	Seropositivity determined at anti-HPV serum cLIA level of ≥ 20, ≥ 16, ≥ 20, or ≥ 24 mMU/mL for HPV6/11/16/18, respectively	A study conducted on a population of sub-Saharan women detected 100% seropositivity in all participants seven months after receiving the 4vHPV vaccine. They found anti-HPV geometric mean titres (GMT) of 602, 626, 3786 and 811 mMU/mL serum levels for HPV 6, 11, 16, 18, respectively, all with a 95% confidence interval.
(28)	4vHPV	Anti-HPV antibodies were used as a geometric mean of MFI; this was assessed via cLIA serology assay and measured with ELISA	An Indian study focused on the effects of mixed dose regimens of 4vHPv administration. They found that regardless of dose frequency, all participants were seropositive 36 months after immunisation. The geometric mean MFIs for HPV16 was 86 for a single dose vaccination, compared to 197 and 196 for two doses and three doses, respectively, three years post vaccination. This indicates a consistency among all three dose variations, albeit with a significant increase in anti-HPV antibodies for the two and three dose schedules.

GMT: geometric mean titre(s); cLIA: competitive Luminex immunoassay; mMU/mL: milli Merck Units/mL; HRHPV: high risk human papilloma virus; LRHPC: low risk human papilloma virus; MFI: mean fluorescence intensity; ELISA: enzyme-linked immunosorbent assay; VLP: virus like particles; IgG: immunoglobulin G; VE: vaccine effectiveness; OR: odds ratio; ≥ CIN2: cervical intraepithelial neoplasia grade 2 or more severe diagnoses; ≥ CIN3: cervical intraepithelial neoplasia grade 3 or more severe diagnoses; RR: relative risk; 95%CI: 95% confidence intervals; IU/mL: international units per millilitre; AU/mL: arbitrary units per millilitre; PCR: polymerase chain reaction; ICC: invasive cervical cancer; CIN3+: intraepithelial neoplasia grade 3. *cervical intraepithelial neoplasia grade 2 or 3, invasive cervical carcinoma and/or adenocarcinoma in situ; º vulvar intraepithelial neoplasia grade 2/3 and/or vulvar cancer; • vaginal intraepithelial neoplasia grade 2/3 and/or vaginal cancer.

## Conclusions

In conclusion, the literature reviewed in this paper have deemed the Gardasil® HPV vaccines to be safe and efficacious. However, it is important to note that among the 30 studies we reviewed, 60% disclosed minor to major involvement with the vaccine manufacturers. Due to the novel nature of these vaccines, long term efficacy is yet to be confirmed as well their associated long-term adverse effects. Nonetheless, many of the studies reported troubling adverse effects, including nausea, fever, abdominal pain, headache and injection site reactions, some of which resulted in prolonged hospitalisations. Considering that these vaccines are predominantly indicated for children, it may be recommended to perform more in-depth analyses on the severity and prevalence of these adverse effects, preferably without the influence of the manufacturers. This is particularly pertinent if these vaccines are to be administered to pregnant women and immunocompromised persons as well. Furthermore, all the studies we investigated reported some level of efficacy, albeit markedly varied in their determinants of efficacy, or endpoints. And thus, a universal value, and more importantly, an agreed definition of efficacy should be implemented, particularly in vaccines that aim to prevent cancer. Equally, more research into the newer 9vHPv should be prioritised as it was only included in approximately a quarter of the studies. In short, studies suggest that the Gardasil® HPV vaccines are generally well tolerated and produce adequate immunity. Even so, the authors of this paper wish to express the importance of further comprehensive, scrupulous and impartial analyzes on these internationally utilised vaccines. Therefore, future research needs to be conducted to ratify the risk-benefit analyzes of these vaccines.
